# Area Vulnerability and Disparities in Therapy for Patients With Metastatic Renal Cell Carcinoma

**DOI:** 10.1001/jamanetworkopen.2024.8747

**Published:** 2024-04-30

**Authors:** Syed N. Rahman, Jessica B. Long, Sarah J. Westvold, Michael S. Leapman, Lisa P. Spees, Michael E. Hurwitz, Hannah D. McManus, Cary P. Gross, Stephanie B. Wheeler, Michaela A. Dinan

**Affiliations:** 1 Department of Urology, Yale School of Medicine, New Haven, Connecticut; 2 Yale Cancer Outcomes, Public Policy, and Effectiveness Research Center, Yale University, New Haven, Connecticut; 3 Department of Health Policy and Management, Gillings School of Global Public Health, The University of North Carolina at Chapel Hill; 4 Lineberger Comprehensive Cancer Center, The University of North Carolina at Chapel Hill; 5 Department of Internal Medicine, Yale School of Medicine, New Haven, Connecticut; 6 Department of Medicine, Duke University School of Medicine, Durham, North Carolina; 7 Department of Chronic Disease Epidemiology, Yale School of Public Health, New Haven, Connecticut

## Abstract

**Importance:**

Area-level measures of sociodemographic disadvantage may be associated with racial and ethnic disparities with respect to receipt of treatment for metastatic renal cell carcinoma (mRCC) but have not been investigated previously, to our knowledge.

**Objective:**

To assess the association between area-level measures of social vulnerability and racial and ethnic disparities in the treatment of US Medicare beneficiaries with mRCC from 2015 through 2019.

**Design, Setting, and Participants:**

This retrospective cohort study included Medicare beneficiaries older than 65 years who were diagnosed with mRCC from January 2015 through December 2019 and were enrolled in fee-for-service Medicare Parts A, B, and D from 1 year before through 1 year after presumed diagnosis or until death. Data were analyzed from November 22, 2022, through January 26, 2024.

**Exposures:**

Five different county-level measures of disadvantage and 4 zip code–level measures of vulnerability or deprivation and segregation were used to dichotomize whether an individual resided in the most vulnerable quartile according to each metric. Patient-level factors included age, race and ethnicity, sex, diagnosis year, comorbidities, frailty, Medicare and Medicaid dual enrollment eligibility, and Medicare Part D low-income subsidy (LIS).

**Main Outcomes and Measures:**

The main outcomes were receipt and type of systemic therapy (oral anticancer agent or immunotherapy from 2 months before to 1 year after diagnosis of mRCC) as a function of patient and area-level characteristics. Multivariable regression analyses were used to adjust for patient factors, and odds ratios (ORs) from logistic regression and relative risk ratios (RRRs) from multinomial logistic regression are reported.

**Results:**

The sample included 15 407 patients (mean [SD] age, 75.6 [6.8] years), of whom 9360 (60.8%) were men; 6931 (45.0%), older than 75 years; 93 (0.6%), American Indian or Alaska Native; 257 (1.7%), Asian or Pacific Islander; 757 (4.9%), Hispanic; 1017 (6.6%), non-Hispanic Black; 12 966 (84.2%), non-Hispanic White; 121 (0.8%), other; and 196 (1.3%), unknown. Overall, 8317 patients (54.0%) received some type of systemic therapy. After adjusting for individual factors, no county or zip code–level measures of social vulnerability, deprivation, or segregation were associated with disparities in treatment. In contrast, patient-level factors, including female sex (OR, 0.78; 95% CI, 0.73-0.84) and LIS (OR, 0.48; 95% CI, 0.36-0.65), were associated with lack of treatment, with particularly limited access to immunotherapy for patients with LIS (RRR, 0.25; 95% CI, 0.14-0.43). Associations between individual-level factors and treatment in multivariable analysis were not mediated by the addition of area-level metrics. Disparities by race and ethnicity were consistently and only observed within the most vulnerable areas, as indicated by the top quartile of each vulnerability deprivation index.

**Conclusions and Relevance:**

In this cohort study of older Medicare patients diagnosed with mRCC, individual-level demographics, including race and ethnicity, sex, and income, were associated with receipt of systemic therapy, whereas area-level measures were not. However, individual-level racial and ethnic disparities were largely limited to socially vulnerable areas, suggesting that efforts to improve racial and ethnic disparities may be most effective when targeted to socially vulnerable areas.

## Key Points

**Question:**

Are area-level measures of social vulnerability associated with disparities in the treatment of US Medicare beneficiaries with metastatic renal cell carcinoma (mRCC)?

**Findings:**

This cohort study of 15 407 individuals with mRCC found no association between area-level measures and treatment disparities after adjusting for individual-level sociodemographics. Racial and ethnic disparities in treatment access appeared to be largely limited to areas with high levels of social vulnerability.

**Meaning:**

In this study, individual-level demographics but not area-level social vulnerability metrics were associated with disparities in receipt of systemic therapy for mRCC.

## Teaser

This cohort study investigates associations between area-level measures of sociodemographic disadvantage and racial and ethnic disparities in receipt of systemic therapy for metastatic renal cell carcinoma among Medicare beneficiaries.

## Introduction

Differential treatment and survival by race and ethnicity have been observed for renal cell carcinoma (RCC) in both the localized and the metastatic setting, and these disparities have persisted with the introduction of novel targeted therapies, such as oral anticancer agents (OAAs) and immunotherapies.^1,2,3^ Racial and ethnic disparities in health reflect structural differences across social factors and resources, including environmental factors and access to health care interventions.^4^ Prevailing evidence suggests that disparities in cancer treatment and mortality associated with individuals’ race and ethnicity are not fully accounted for by individual-level, clinical, and demographic factors and that neighborhood factors influencing health and health care access could be contributors.^5,6,7,8^

There has been growing interest in how area-level measures of socioeconomic status and environment are associated with health outcomes. Area-level measures have been associated with frailty, obesity, and cardiovascular disease as well as access to preventive health care in historically marginalized populations.^4,5,6,9,10^ In regard to cancer-specific outcomes, a higher Social Vulnerability Index (SVI)^11^ score has been associated with patients being less likely to receive neoadjuvant chemotherapy or to use high-volume centers for surgical resection and with patients being more likely to encounter fragmented postoperative care.^7,8,11,12^ Additionally, area-level factors have been associated with morbidity after controlling for sociodemographic factors among individuals with colorectal and breast cancer.^13^

Metrics that are commonly used to quantify social determinants of health at the county and zip code level include the Social Deprivation Index (SDI)^12^ and Index of Concentration at the Extremes (ICE), a measure of spatial social polarization that quantifies racial residential segregation.^13^ These measures incorporate both the social and the built environment of the community and include socioeconomic status, educational level, housing quality, transportation, health infrastructure, language barriers, and medical vulnerability at the area level. Prior research demonstrates that these area-level measures are independently associated with cancer incidence, screening, treatment, and outcomes in both localized and metastatic cancers, showing that the community within which patients live can have consequences for their health.^14,15,16^

Prior literature has demonstrated a compounding negative association between social vulnerability and individual-level measures and disparities in survival for common, largely treatable cancers.^17,18,19^ The investigation of possible mediating effects of area-level metrics may therefore help explain treatment and potentially survival differences. A previous analysis of Medicare beneficiaries with metastatic RCC (mRCC) identified disparities in the receipt of immunotherapies and OAAs associated with individuals’ race.^3^ However, that analysis did not explore the complex relationship between individual-level measures of socioeconomic status (SES), area-level measures of SES and vulnerability, and treatment receipt.^20^

In this study, we examined available social indices of social determinants of health, including SVIs, SDIs, and ICE measures; examined their association with disparities in mRCC treatment after adjusting for patient-level factors; and further incorporated patient-level SES metrics in the form of LIS eligibility.^15^ Our objectives were to assess whether area-level measures of disadvantage were independently associated with adoption of emerging therapies and to understand how they interact with race and ethnicity to impact receipt of treatment.

## Methods

### Study Population

We conducted a retrospective cohort study of fee-for-service Medicare beneficiaries aged 66 years or older enrolled in Medicare Parts A, B, and D who were diagnosed with mRCC from January 2015 through December 2019. Patients who died less than 1 month after diagnosis were excluded. We identified the earliest systemic therapy a patient received using health insurance claims in the 2 months before through 12 months after presumed diagnosis. This study was determined to be exempt by the Yale School of Medicine Human Investigation Committee, including waivers of informed consent and Health Insurance Portability and Accountability Act authorization, because it used secondary data for research purposes. We followed the Strengthening the Reporting of Observational Studies in Epidemiology (STROBE) reporting guideline; however, some of this information was previously published.^3^ We identified contemporary US Food and Drug Administration–approved OAA agents using generic names and immunotherapy agents using Healthcare Common Procedure Coding System codes. Additional details are provided in the eMethods in Supplement 1.

### Area-Level Metrics of Vulnerability

We identified indexed area-level metrics (Box) based on US census and American Community Survey data and linked them at the county level using Federal Information Processing Standards state and county codes for the year of diagnosis for patients in the cohort or 5-digit zip codes. Though each measure is distinct, we use the general term *vulnerability* to refer to these metrics hereafter.

Box. **Input Parameters of Area Factors**

**Socioeconomic Status**

Below poverty level (SVI, MH SVI, and SDI)
Unemployed (SVI, MH SVI, and SDI)
Income (SVI, MH SVI, and income segregation)
No high school diploma (SVI, MH SVI, and education segregation)

**Household Composition and Disability**

Age ≥65 y (SVI, MH SVI)
Age ≤17 y (SVI, MH SVI)
Age >5 y with a disability (SVI, MH SVI)
Single-parent household (SVI, MH SVI, and SDI)

**Housing Type and Transportation**

Multiunit structure (SVI, MH SVI)
Mobile home (SVI, MH SVI)
Crowding (SVI, MH SVI, and SDI)
No vehicle (SVI, MH SVI, SDI)
Group quarters (SVI, MH SVI)
Rental housing (SDI)

**Minority Status and Language**

Any minority group (SVI)
Anyone speaking English less than very well (SVI)
African American or non-Hispanic Black individuals (MH SVI, SDI, segregation, income segregation, and education segregation)
Other minority group (MH SVI): American Indian or Alaska Native, Asian, Native Hawaiian or Pacific Islander, Hispanic or Latinx, and other race or ethnicity
Groups who speak English less than very well (MH SVI): Spanish, Chinese, Korean, Vietnamese, and Russian

**Health Care Infrastructure and Access**

Hospitals, urgent care clinics, pharmacies, primary care physicians, and health insurance (MH SVI)

**Medical Vulnerability**

Cardiovascular disease, chronic respiratory disease, obesity, diabetes, and internet access (MH SVI)

Abbreviations: MH SVI, Minority Health Social Vulnerability Index; SDI, Social Deprivation Index; SVI, Social Vulnerability Index.

### Statistical Analysis

We examined the distribution of the area vulnerability measures as continuous constructs within the sample, categorized each area factor into quartiles, and compared the most vulnerable quartile with the other quartiles. We examined the use of immunotherapies, OAAs, or other systemic therapies as initial treatment for mRCC from 2015 to 2019 according to categorized area factors, using χ^2^ tests to compare receipt of any treatment separately from type of treatment.

We used logistic regression to model receipt of any systemic therapy compared with no therapy in the 2 months before through the 12 months after diagnosis or until death and report odds ratios (ORs) associated with receipt of any treatment. To assess the type of treatment received (OAA vs immunotherapy vs other), we used multinomial logistic regression with no treatment as the reference group. We assessed unadjusted and adjusted associations of each area vulnerability factor, including year of diagnosis, race and ethnicity, sex, age at diagnosis, Elixhauser Comorbidity Index score,^21,22^ Kim claims-based frailty index score,^23^ metropolitan residential status, Medicare and Medicaid dual eligibility, and Medicare Part D low-income subsidy (LIS) eligibility as independent individual-level variables. Race and ethnicity were ascertained by the Social Security Administration and revised by the Research Triangle Institute imputation algorithm to improve accuracy for identification of Asian or Pacific Islander and Hispanic beneficiaries; categories were American Indian or Alaska Native, Asian or Pacific Islander, Hispanic, non-Hispanic Black (hereafter, *Black*), non-Hispanic White (hereafter, *White*), and other (directly reported as such) or unknown. For the multinomial regression regarding type of treatment, we report adjusted relative risk ratios (RRRs). We report specific estimates only for Black and Hispanic compared with White race and ethnicity based on prior analyses demonstrating that these groups had statistically different receipt of therapy.^3^ We used SAS, version 9.4 (SAS Institute Inc) for data management, Stata, release 18 (StataCorp LLC) for statistical analysis, and GraphPad Prism 9 for graphical display of the findings. Data were analyzed from November 22, 2022, through January 26, 2024. Two-sided *P* < .05 was considered significant.

Our initial hypothesis was that area-level metrics would be associated with access to recently approved immunotherapy. However, we observed no evidence of such an association after adjusting for individual-level factors. To further explore this null result, we conducted post hoc mediation and moderation analyses to assess the ability of area-level factors to modulate and attenuate, respectively, the association with individual demographic factors. Specifically, we conducted mediation analysis by comparing individual-level demographic coefficients in models that did vs did not include adjustment by SVI metrics. We then conducted a moderation analysis by comparing patient-level variable coefficients between SVI quartiles to examine whether individual-level disparities might be moderated by area-level environments. Fewer than 11 patients were available for nearly 90% of included counties and more than 99% of zip codes. Attempting to incorporate fixed or random effects at higher levels would have widened 95% CIs around an already null association between area-level factors and treatment.

## Results

We identified 15 407 patients who met the sample inclusion criteria (mean [SD] age, 75.6 [6.8] years), of whom 6931 (45.0%) were older than 75 years; 9360 (60.8%), men; 6047 (39.2%), women; 93 (0.6%), American Indian or Alaska Native; 257 (1.7%), Asian or Pacific Islander; 1017 (6.6%), Black; 757 (4.9%), Hispanic; 12 966 (84.2%), White; 121 (0.8%), other; and 196 (1.3%), unknown. Across all years combined, 8317 patients (54.0%) received some type of systemic therapy. Overall, 3630 (23.6%) received OAA, 2553 (16.6%) received immunotherapy, and 2134 (13.9%) received other systemic treatment. As reported in our prior analysis,^3^ the proportion of patients receiving any systemic therapy increased from 1511 of 2951 (51.2%) in 2015 to 1886 of 3185 (59.2%) in 2019, primarily due to uptake of immunotherapy, which was greatest among White patients. Patient-level factors, including female sex (OR, 0.78; 95% CI, 0.73-0.84) and LIS (OR, 0.48; 95% CI, 0.36-0.65), were associated with reduced receipt of treatment, with particularly limited access to immunotherapy for patients with LIS (RRR, 0.25; 95% CI, 0.14-0.43).

### LIS and Treatment Utilization

The greatest disparities in treatment receipt and type of treatment were associated with individual-level surrogates for low income, including receipt of LIS and Medicaid dual enrollment. Only 1356 of 2959 patients with LIS (45.8%) received any treatment compared with 6961 of 12 448 individuals without LIS (55.9%), and only 278 patients with LIS (9.4%) received immunotherapy compared with 2275 without LIS (18.3%) (*P* < .001). Medicaid dual enrollment was also associated with decreased receipt of any treatment between 2015 and 2019 (ranging from 249 of 538 [46.3%] to 196 of 442 [44.3%] per year) compared with ineligibility, which was associated with increased receipt (1267 of 2413 [52.5%] to 1693 of 2743 [61.7%] per year). For immunotherapy, dual eligibility was associated with decreased receipt of treatment in 2019 (97 of 442 patients [21.9%]) compared with ineligibility (1090 of 2743 [39.7%]). Additionally, compared with those with a premium, fewer patients with a $0 premium and co-payment received any treatment (41 of 136 [30.1%] vs 1654 of 2673 [61.9%] in 2019) or treatment with immunotherapy (17 [12.5%] vs 1069 [40.0%] in 2019). Individuals without Medicaid dual eligibility or those who had more than a $0 Part D premium responsibility had similar treatment receipt over time.

### Association of Area-Level Factors of Vulnerability With Treatment Utilization

**Figure 1.**
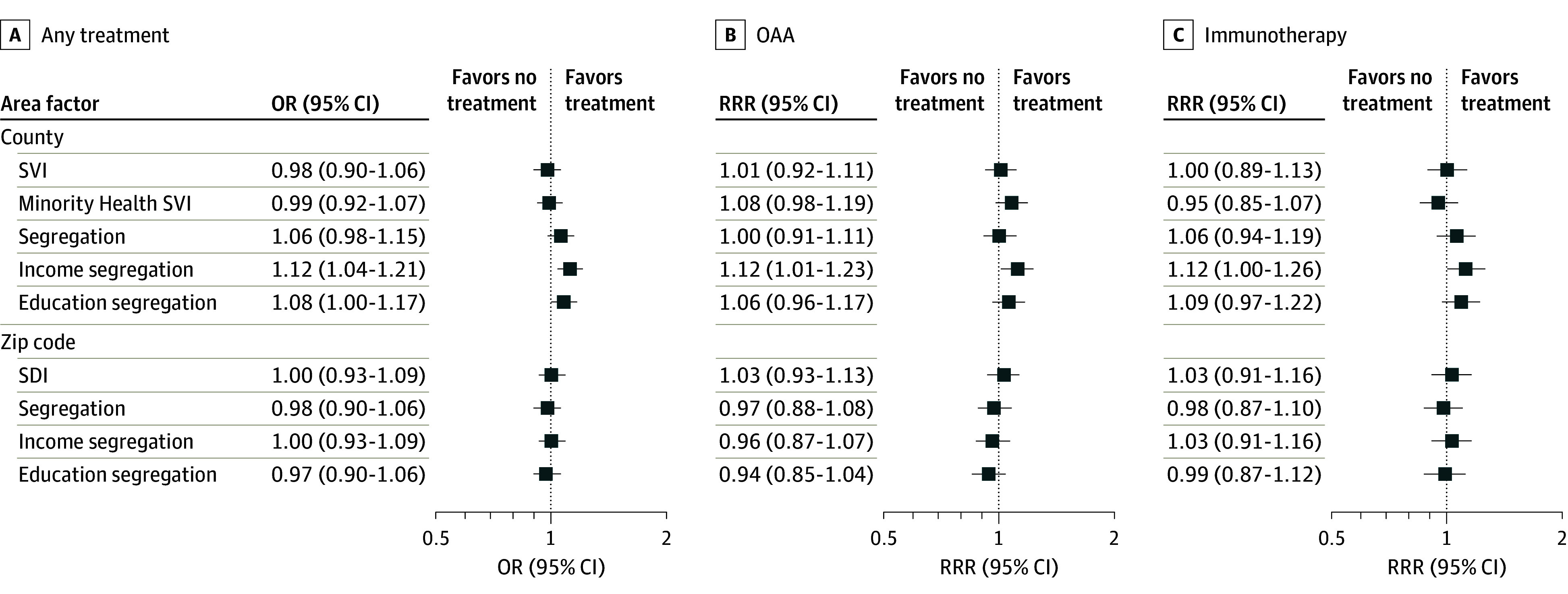
Forest Plot of Adjusted Associations Between Area Factors and Treatment Receipt for the Most Vulnerable Quartile Compared With the Other Quartiles Adjusted for year of diagnosis, race and ethnicity, sex, age at diagnosis, Elixhauser Comorbidity Index score, Kim claims-based frailty index score, metropolitan residential status, Medicare and Medicaid dual eligibility, and Medicare Part D low-income subsidy eligibility as independent individual-level variables. OAA indicates oral anticancer agent; OR, odds ratio; RRR, relative risk ratio; SDI, Social Deprivation Index; SVI, Social Vulnerability Index.

At the zip code level and county level, associations between area-level metrics and receipt of treatment were observed. The number of beneficiaries available for each model varied slightly according to those who could be linked to the area-level metrics (eTable 1 in Supplement 1). However, in fully adjusted models, only income segregation comparing the most deprived quartile with the others demonstrated a persistent association with greater odds of receiving any treatment (OR, 1.12; 95% CI, 1.04-1.21) and OAA receipt (RRR, 1.12; 95% CI, 1.01-1.23) (Figure 1). No additional area measures (SVI, Minority Health SVI, SDI, or zip code–level segregation) were associated with receipt of any treatment or a specific type of treatment.

### Mediation of Association Between Race and Ethnicity and Treatment Receipt by Area-Level Factors of Vulnerability

**Figure 2.**
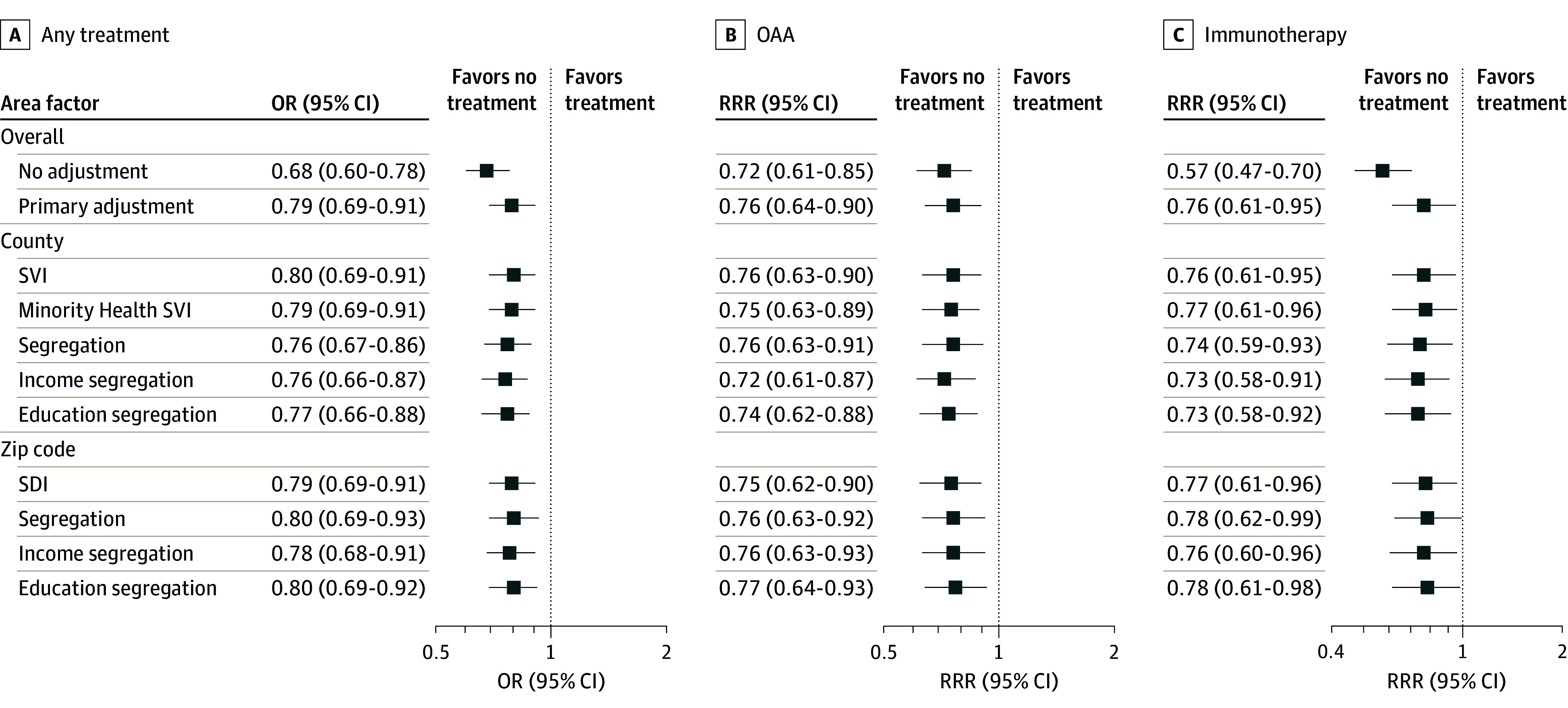
Forest Plot of Associations Between Race and Treatment Receipt Among Non-Hispanic Black and Non-Hispanic White Patients Adjusted for area variables (county-level measures: Social Vulnerability Index [SVI], Minority Health SVI, segregation, income segregation, and education segregation; zip code–level measures: Social Deprivation Index [SDI], segregation, income segregation, and education segregation) and patient variables (index year, age group, sex, Elixhauser Comorbidity Index group, frailty, metropolitan residence, dual eligibility, and low-income subsidy). OAA indicates oral anticancer agent; OR, odds ratio; RRR, relative risk ratio.

**Figure 3.**
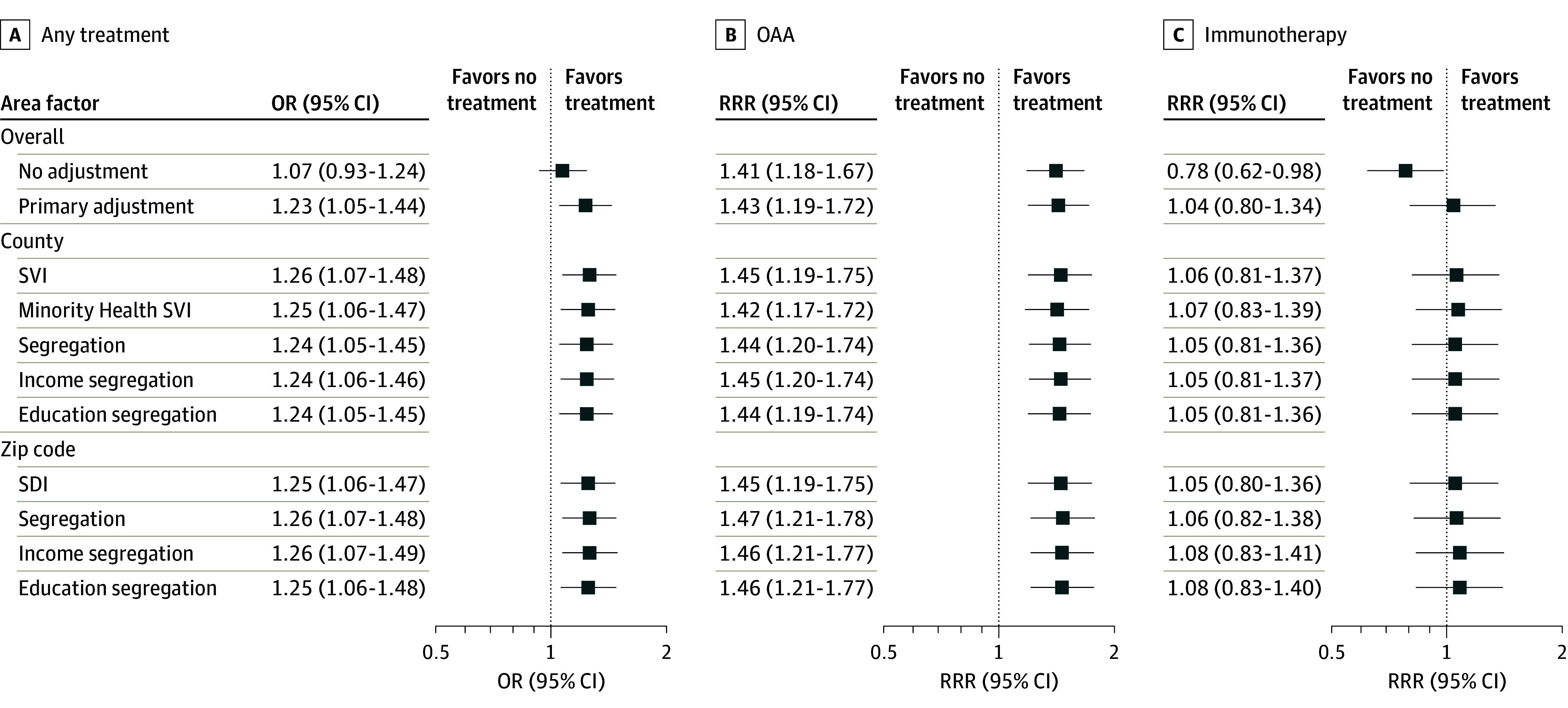
Forest Plot of Associations Between Ethnicity and Treatment Receipt Among Hispanic and Non-Hispanic White Patients Adjusted for area variables (county-level measures: Social Vulnerability Index [SVI], Minority Health SVI, segregation, income segregation, and education segregation; zip code–level measures: Social Deprivation Index [SDI], segregation, income segregation, and education segregation) and patient variables (index year, age group, sex, Elixhauser Comorbidity Index group, frailty, metropolitan residence, dual eligibility, and low-income subsidy). OAA indicates oral anticancer agent; OR, odds ratio; RRR, relative risk ratio.

Black compared with White race and ethnicity was associated with lower rates of any treatment (OR, 0.79; 95% CI, 0.69-0.91), OAA receipt (RRR, 0.76; 95% CI, 0.63-0.90), and immunotherapy receipt (RRR, 0.76; 95% CI, 0.61-0.95) when adjusting for other patient characteristics (Figure 2). Additionally, Hispanic individuals compared with White individuals had higher odds of receipt of any treatment (OR, 1.23; 95% CI, 1.05-1.44) and OAA treatment (RRR, 1.43; 95% CI, 1.19-1.72), but there was no difference in receipt of immunotherapy (RRR, 1.04; 95% CI, 0.80-1.34) (Figure 3). These associations did not change after adding any area vulnerability to the models, suggesting that area vulnerability was not directly mediating any of these observed individual-level associations.

### Moderation of Disparities by Area-Level Factors

In our multivariable analyses, we did not observe a significant association between treatment receipt and area-level factors after adjusting for individual-level factors. However, we next investigated whether the magnitude of individual-level disparities was moderated by high vs low areas of social vulnerability. We hypothesized that individual-level disparities might be most pronounced in the most vulnerable areas. To test this hypothesis, we applied our multivariable models to the overall cohort and then compared the observed outcomes when analyzed within quartiles of SVIs.

Comparing the most vs least vulnerable quartiles of SVIs (3881 and 3795 patients, respectively) revealed that the most vulnerable areas had higher proportions of Black patients (423 [10.9%] vs 122 [3.2%]) and patients with 3 or more comorbidities (1468 [37.8%] vs 1270 [33.5%]), likely to have frailty (1368 [35.2%] vs 1130 [29.8%]), with dual eligibility (1023 [26.4%] vs 415 [10.9%]), and with residence within a metropolitan area (2581 [66.5%] vs 3016 [79.5%]) (all *P* < .001) (eTable 2 in Supplement 1). Unlike in the overall cohort, receipt of any treatment was not associated with Black race or Hispanic ethnicity within the least vulnerable counties when examined using any of the county- or area-level metrics, including Centers for Disease Control and Prevention (CDC) SVI (Table), Office of Minority Health SVI, SDI, county ICE segregation, or zip code ICE segregation. In contrast, differences in receipt of treatment by race and ethnicity were only observed in the most and second most vulnerable quartiles of area-level measures. For example, Black race was not associated with receipt of any treatment in the least-vulnerable quartile by CDC SVI (OR, 1.15; 95% CI, 0.79-1.69) but was associated with lower rates of any treatment in the second most vulnerable (OR, 0.69 95% CI, 0.54-0.88) and most vulnerable (OR, 0.80; 95% CI, 0.64-1.00) quartiles. Disparities in receipt of treatment by LIS and sex, however, were comparable across quartiles of social vulnerability, suggesting that racial and ethnic disparities were most prominent in socially vulnerable areas, whereas income and sex disparities were not moderated by area-level social vulnerability. Analysis of treatment type (OAA vs none and immunotherapy vs none) showed a similar trend (eTables 3 and 4 in Supplement 1), but statistical power in the immunotherapy analysis was limited by small sample sizes.

**Table.** Adjusted Logistic Regression for Receipt of Any Treatment, Stratified by CDC SVI Quartile

Characteristic	Receipt of any treatment, AOR (95% CI)			
	Quartile 1 (least vulnerable)	Quartile 2	Quartile 3	Quartile 4 (most vulnerable)
Unadjusted proportion receiving any treatment, %	56	54	54	53
Patient race and ethnicity				
American Indian or Alaska Native, other, or unknown	0.92 (0.61-1.37)	1.10 (0.70-1.71)	0.88 (0.58-1.32)	1.22 (0.80-1.84)
Asian or Pacific Islander	1.25 (0.67-2.32)	1.05 (0.68-1.64)	1.55 (0.84-2.86)	0.96 (0.58-1.59)
Hispanic	0.90 (0.51-1.56)	1.31 (0.86-2.01)	1.34 (0.94-1.89)	1.29 (1.02-1.63)
Non-Hispanic Black	1.15 (0.79-1.69)	0.80 (0.56-1.16)	0.69 (0.54-0.88)	0.80 (0.64-1.00)
Non-Hispanic White	1 [Reference]	1 [Reference]	1 [Reference]	1 [Reference]
Dual eligible	1.08 (0.62-1.91)	1.05 (0.60-1.84)	0.84 (0.54-1.31)	0.91 (0.58-1.41)
Low-income subsidy				
0%-75% Premium subsidy	1 [Reference]	1 [Reference]	1 [Reference]	1 [Reference]
100% Premium subsidy, no co-payment	0.53 (0.27-1.04)	0.39 (0.20-0.77)	0.55 (0.31-0.97)	0.47 (0.28-0.79)
100% Premium subsidy, any co-payment	0.79 (0.47-1.33)	0.91 (0.54-1.55)	0.90 (0.60-1.36)	0.95 (0.62-1.45)
Year of mRCC diagnosis				
2015	1 [Reference]	1 [Reference]	1 [Reference]	1 [Reference]
2016	1.04 (0.83-1.30)	0.97 (0.77-1.22)	1.01 (0.80-1.27)	1.06 (0.84-1.32)
2017	1.34 (1.06-1.68)	1.32 (1.04-1.67)	1.21 (0.96-1.53)	1.34 (1.06-1.68)
2018	1.64 (1.29-2.07)	1.68 (1.34-2.12)	1.58 (1.25-2.00)	1.48 (1.18-1.87)
2019	1.90 (1.50-2.39)	1.54 (1.22-1.94)	1.57 (1.24-1.98)	1.77 (1.40-2.24)
Age at diagnosis, y				
66-70	1 [Reference]	1 [Reference]	1 [Reference]	1 [Reference]
71-75	0.94 (0.79-1.13)	0.94 (0.78-1.12)	0.96 (0.80-1.15)	0.93 (0.78-1.11)
76-80	0.89 (0.74-1.08)	0.79 (0.65-0.95)	0.84 (0.69-1.01)	0.86 (0.71-1.04)
≥81	0.48 (0.40-0.58)	0.44 (0.36-0.53)	0.40 (0.34-0.49)	0.49 (0.40-0.59)
Female	0.74 (0.65-0.85)	0.72 (0.63-0.82)	0.83 (0.73-0.95)	0.83 (0.73-0.95)
Comorbidity conditions, No.				
None	1 [Reference]	1 [Reference]	1 [Reference]	1 [Reference]
1-2	0.90 (0.75-1.08)	0.85 (0.71-1.02)	0.93 (0.77-1.12)	0.92 (0.76-1.11)
≥3	0.82 (0.67-1.02)	0.68 (0.55-0.84)	0.85 (0.68-1.05)	0.80 (0.64-0.99)
Likely to have frailty	0.79 (0.66-0.94)	0.77 (0.65-0.92)	0.79 (0.66-0.94)	0.78 (0.66-0.93)
Patient lives in metropolitan area	0.95 (0.81-1.12)	1.04 (0.89-1.23)	1.00 (0.84-1.18)	0.94 (0.82-1.08)

Abbreviations: AOR, adjusted odds ratio; CDC SVI, Centers for Disease Control and Prevention Social Vulnerability Index; mRCC, metastatic renal cell carcinoma.

## Discussion

This study investigated the association between area-level factors and treatment utilization in a nationally representative population of patients with mRCC following the approval of checkpoint immmunotherapy in 2015. We found that although area-level metrics of social vulnerability were associated with treatment utilization in unadjusted analyses, these associations disappeared after adjusting for patient-level factors. Consistent with our previously published work,^3^ we identified that Black race was associated with lower rates of OAA and immunotherapy receipt. Contrary to our hypothesis, we did not observe consistent associations between county- and zip code–level area measures of vulnerability and receipt of systemic therapies after adjusting for individual-level factors, and the inclusion of area-level factors did not mitigate individual-level factors associated with lower rates of treatment, including sex, race, ethnicity, or low income. However, when examining racial and ethnic disparities by least vs most vulnerable areas, we found that these disparities predominantly occurred within the context of socially vulnerable areas. In contrast, differences in treatment associated with low income, sex, age, or comorbidities were similar across all quartiles of area vulnerability.

Our study demonstrated no direct association between area-level factors and treatment utilization for mRCC. Studies of differences in cancer treatment have not included RCC and have largely been in the surgical setting, evaluating whether area-level factors are associated with differences in receipt of cancer surgery.^23,24^ Prior work that focused on neighborhood characteristics showed neighborhood-level associations with disparities in stage at diagnosis and outcomes for common cancers such as lung, breast, and colorectal cancer but did not examine treatment.^18,19,25^ The findings of these studies may be less generalizable to a metastatic setting, for which treatment plans are more individualized and survival rates are lower. One study of patients with metastatic cancer that used medical and pharmacy claims data showed that individuals from areas with increased vulnerability (SVI) were up to 30% less likely to be treated within 30 days after a metastatic cancer diagnosis.^15^ Our study used a horizon of 1 year and may not have detected disparities in delayed initiation of care.

Although a direct association between vulnerable areas and treatment did not persist in adjusted analyses, we found that disparities in individuals’ treatment associated with their race appeared to occur uniquely within economically vulnerable areas, suggesting that these at-risk areas harbor substantially higher rates of disparities. We observed that treatment disparities associated with race and ethnicity were confined to the most vulnerable areas. However, these areas were also enriched for racial and ethnic minority populations, which could explain in part why disparities were observed in these areas. Nonetheless, point estimates of less vulnerable areas were closer to unity (no effect), which supports the lack of an observed disparity as opposed to merely being underpowered. This idea of more vulnerable areas having exaggerated disparities would not necessarily result in a direct association in logistic modeling but nonetheless can be considered as a moderator of the association between disparities and treatment receipt. This is consistent with prior literature that has demonstrated associations between areas with a higher SVI and increased likelihood of racial disparities and insurance disparities among surgical patients.^26,27,28^

### Limitations

This study has limitations. Factors such as affordability, transportation, and social or logistic support may all present major barriers to care but were not examined in this study. Claims data do not provide information on individuals’ disease characteristics, such as burden of metastatic disease, sites of metastases, and histologic features, which may explain some treatment differences. Although immunotherapy treatment for RCC was approved in 2015, it was not approved in the first-line setting until 2018, which limits evaluation of first-line immunotherapy use in our study period (2015-2019).^2^ We did not know a person’s individual income, only if they were dually Medicare-Medicaid enrolled and the type of LIS they received. Consequently, we could not capture patients with low income above the Medicaid and low-income coverage limits.

We examined area-level factors at the level of county and zip code, which may have missed features at higher levels of resolution, such as census tract. Zip code was the smallest geographic unit available in the source data; however, census tract–level metrics for the vulnerability measures may be a subject of future research in alternate data sources. Previous studies have found associations between county- and zip code–level factors and disparities in health care access.^7,8,11^

Another limitation is that using Medicare fee-for-service is limited to older patients and may not be generalizable to patients younger than 66 years. Our study examined only Medicare fee-for-service beneficiaries, and the association of area-level resources with disparities in patients with Medicare Advantage (MA) cannot be commented on. Patients with MA in general tend to be healthier and perhaps more likely to be eligible for aggressive therapy but conversely may also be hindered by potential limitations to care, including details of MA plan features and the requirement to seen by in-network health care professionals and associated facilities.

## Conclusions

In this retrospective cohort study of Medicare beneficiaries diagnosed with mRCC between 2015 and 2019, we observed differences by race and ethnicity in receipt of systemic therapies. We did not observe a direct association between area-level measures of disadvantage and receipt of treatment but found that racial and ethnic treatment disparities appeared to occur largely within socially vulnerable areas. Race and ethnicity, limited-income subsidy, and dual Medicaid enrollment were associated with decreased access to systemic therapy. Our findings support the use of interventions to reduce the effects of social determinants of health on care disparities for patients with metastatic cancer within at-risk areas but directed at the patient level. Future research is warranted to explore receipt of emerging advanced therapies for metastatic cancer to help guide interventions to provide more equitable care.

## Notes

Supplement 1.
eMethods.
**eTable 1.** Area-Level Measure Descriptive Statistics **eTable 2.** Demographics of Study Cohort Overall and Stratified by Quartile of CDC Social Vulnerability Index **eTable 3.** Oral Anticancer Agent Treatment vs No Treatment, Stratified by CDC SVI Quartile **eTable 4.** Immunotherapy Treatment vs No Treatment, Stratified by CDC SVI Quartile
eReferences

Supplement 2.
Data Sharing Statement
