# Supplementary material for: Area Vulnerability and Disparities in Therapy for Patients With Metastatic Renal Cell Carcinoma
Source: JAMA Netw Open. 2024 Apr 30;7(4):e248747. doi: 10.1001/jamanetworkopen.2024.8747 (PMC11061765; doi:10.1001/jamanetworkopen.2024.8747)
Supplement: Supplement 1. — eMethods. eTable 1. Area-Level Measure Descriptive Statistics eTable 2. Demographics of Study Cohort Overall and Stratified by Quartile of CDC Social Vulnerability Index eTable 3. Oral Anticancer Agent Treatment vs No Treatment, Stratified by CDC SVI Quartile eTable 4. Immunotherapy Treatment vs No Treatment, Stratified by CDC SVI Quartile eReferences [file jamanetwopen-e248747-s001.pdf]

# Supplementary Online Content

Rahman SN, Long JB, Westvold SJ, et al. Area vulnerability and disparities in therapy for patients with metastatic renal cell carcinoma. *JAMA Netw Open*. 2024;7(4):e248747. doi:10.1001/jamanetworkopen.2024.8747

**eMethods.**
**eTable 1.** Area-Level Measure Descriptive Statistics
**eTable 2.** Demographics of Study Cohort Overall and Stratified by Quartile of CDC Social Vulnerability Index
**eTable 3.** Oral Anticancer Agent Treatment vs No Treatment, Stratified by CDC SVI Quartile
**eTable 4.** Immunotherapy Treatment vs No Treatment, Stratified by CDC SVI Quartile
**eReferences**

This supplementary material has been provided by the authors to give readers additional information about their work.

© 2024 Rahman SN et al. *JAMA Network Open*.

**eMethods**

We identified contemporary FDA-approved OAA agents using generic names for sorafenib, sunitinib, pazopanib, everolimus, axitinib, lenvatinib mesylate, and cabozantinib s-malate. Our dataset included claims through December 31, 2020. No claims indicating tivozanib hydrochloride were identified. We identified the following IO agents using Healthcare Common Procedure Codes (HCPCS) ipilimumab (C9284, J9228), nivolumab (C9453, J9299), pembrolizumab (C9027, J9271), avelumab (C9491, J9023), interleukin-2 or aldesleukin (J9015), and interferon-alfa (J9212, J9213, J9214, J9215). Patients who received both an OAA and IO as their earliest therapies within 60 days of the other were assigned to the IO group for analysis, since IO receipt was considered the most novel agent for which we suspected the greatest disparity in access. Additional therapies received were identified, including: temsirolimus (J9330), bevacizumab (J9035), systemic agents (Q0083-Q0085, G0355-G0363, J8510-J9999), and claims indicating chemotherapy administration (96400-96549).

*Patient-Level Covariates*

Patient-level variables included: year of diagnosis, race-ethnicity,[1] sex, age at diagnosis, Elixhauser comorbidity score[2], and Kim claims-based frailty index.[3]. We categorized race-ethnicity as American Indian/Native Alaskan/Other/Unknown (combined due to small cells with n<11), Asian/Pacific Islander, Hispanic, non-Hispanic Black (NHB) or non-Hispanic White (NHW). We used the Research Triangle Institute Race Code variable from the Medicare database which has employed an imputation algorithm to improve accuracy for identification of Hispanic, Asian/Pacific Islander beneficiaries[1]. Medicare/Medicaid dual-enrollment and Part D Low Income Subsidy (LIS) eligibility were used as patient-level surrogates for low-income status. Part D LIS is a program that provides cost-sharing support for Part D prescription drugs for Medicare beneficiaries with limited

© 2024 Rahman SN et al. *JAMA Network Open*.

incomes (<150% federal poverty level) leading to varying levels of premium and copay as low as $0.[4] As previously described in the mRCC setting, dual eligibility has been associated with poor treatment initiation in the elderly population, thus leading us to investigate the association between LIS and dual eligibility status and treatment receipt in the mRCC population as a whole.[5] We also include metropolitan status based on the patient's county of residence as an individual-level predictor.

Area-Level Metrics of Vulnerability

We identified the following indexed area-level metrics (**Table 1**) based on census and American Community Survey (ACS) data and linked them at county level using Federal Information Processing System State and county codes for the year of diagnosis for patients in our cohort or five-digit zip code. County indexed metrics included (1) Center for Disease Control/Agency for Toxic Substance and Disease Registry Social Vulnerability Index (SVI) incorporating factors such as socioeconomic status, household composition and disability, housing type and transportation, minority status and language[6], and (2) Office of Minority Health SVI (MH SVI) building on the prior CDC SVI with more specific minority and language categorization and variables on health care infrastructure and medical vulnerability[7]. At the zip code level, we included Graham Institutes Social Deprivation Index (SDI)[8], which captures similar components as the county SVI measures. Additionally, we used data from the ACS for 2015-2019 accessed through National Historical Geographic Information System (NHGIS) to calculate Index of Concentration at Extremes (ICE) measures of racial segregation, racial income segregation, and racial education segregation. These metrics have been previously described by Krieger et al. in 2017 and compare NHB and NHW residents at both the county and five digit zip code levels.[9] For SVI, MH SVI, and SDI a higher value indicates higher vulnerability. For ICE values range from -1 to +1 where -1

© 2024 Rahman SN et al. *JAMA Network Open*.

indicates an area only including vulnerable individuals. Though each measure is distinct we will use the terminology of ‘vulnerability’ to generally refer to these metrics moving forward in the manuscript.

© 2024 Rahman SN et al. *JAMA Network Open*.

**eTable 1** – Area Level Measure Descriptive Statistics

| | N | Mean (Standard Deviation) | Median (Quartile 1-Quartile 3) |
|---|---|---|---|
| **County Linked Measures** | | | |
| Social Vulnerability Index (SVI) | 15367 | 51 (26) | 52 (29-72) |
| Minority Health SVI | 15367 | 61 (26) | 63 (41-84) |
| Segregation (ICE) | 15367* | 0.70 (0.30) | 0.81 (0.54-0.94) |
| Income Segregation (ICE) | 15367 | 0.64 (0.39) | 0.79 (0.47-0.93) |
| Education Segregation (ICE) | 15367* | 0.35 (0.46) | 0.42 (0.00-0.75) |
| **Zip Code Linked Measures** | | | |
| Social Deprivation Index | 15165 | 45 (27) | 43 (22-67) |
| Segregation (ICE) | 15165* | 0.73 (0.39) | 0.89 (0.68-0.97) |
| Income Segregation (ICE) | 15146 | 0.38 (0.56) | 0.57 (0.01-0.83) |
| Education Segregation (ICE) | 15078 | 0.66 (0.51) | 0.91 (0.56-1.00) |

* True value +/- 10 from shown value due to CMS policy of not publishing cells or calculable fields n<11.

© 2024 Rahman SN et al. *JAMA Network Open*.

**eTable 2 -** Demographics of study cohort overall and stratified by quartile of CDC Social Vulnerability Index

| **Characteristic** | Total | | Quartile 1 (Least Vulnerable) | | Quartile 2 | | Quartile 3 | | Quartile 4 (Most Vulnerable) | |
|---|---|---|---|---|---|---|---|---|---|---|
| | N | % | N | % | N | % | N | % | N | % |
| | 15,367 | | 3,795 | | 3,885 | | 3,806 | | 3,881 | |
| **Year of metastatic diagnosis** | | | | | | | | | | |
| 2015 | 2,944 | 19.2 | 722 | 19.0 | 701 | 18.0 | 730 | 19.2 | 791 | 20.4 |
| 2016 | 3,067 | 20.0 | 765 | 20.2 | 773 | 19.9 | 777 | 20.4 | 752 | 19.4 |
| 2017 | 3,114 | 20.3 | 776 | 20.4 | 753 | 19.4 | 781 | 20.5 | 804 | 20.7 |
| 2018 | 3,072 | 20.0 | 732 | 19.3 | 846 | 21.8 | 733 | 19.3 | 761 | 19.6 |
| 2019 | 3,170 | 20.6 | 800 | 21.1 | 812 | 20.9 | 785 | 20.6 | 773 | 19.9 |
| **Patient Race and Ethnicity** | | | | | | | | | | |
| American Indian / Alaska Native, Other, Unknown | 408 | 2.7 | 109 | 2.9 | 94 | 2.4 | 103 | 2.7 | 102 | 2.6 |
| Asian/Pacific Islander | 257 | 1.7 | 46 | 1.2 | 92 | 2.4 | 47 | 1.2 | 72 | 1.9 |
| Hispanic | 741 | 4.8 | 55 | 1.4 | 102 | 2.6 | 155 | 4.1 | 429 | 11.1 |
| Non-Hispanic Black | 1,013 | 6.6 | 122 | 3.2 | 136 | 3.5 | 332 | 8.7 | 423 | 10.9 |
| Non-Hispanic White | 12,948 | 84.3 | 3,463 | 91.3 | 3,461 | 89.1 | 3,169 | 83.3 | 2,855 | 73.6 |
| **Age at diagnosis** | | | | | | | | | | |
| 66-70 | 4,251 | 27.7 | 1,034 | 27.2 | 1,078 | 27.7 | 1,048 | 27.5 | 1,091 | 28.1 |
| 71-75 | 4,208 | 27.4 | 1,049 | 27.6 | 1,041 | 26.8 | 1,022 | 26.9 | 1,096 | 28.2 |
| 76-80 | 3,297 | 21.5 | 809 | 21.3 | 840 | 21.6 | 832 | 21.9 | 816 | 21.0 |
| 81+ | 3,611 | 23.5 | 903 | 23.8 | 926 | 23.8 | 904 | 23.8 | 878 | 22.6 |
| **Patient sex** | | | | | | | | | | |
| Male | 9,332 | 60.7 | 2,345 | 61.8 | 2,394 | 61.6 | 2,285 | 60.0 | 2,308 | 59.5 |
| Female | 6,035 | 39.3 | 1,450 | 38.2 | 1,491 | 38.4 | 1,521 | 40.0 | 1,573 | 40.5 |
| **Comorbidity score** | | | | | | | | | | |
| No conditions | 4,813 | 31.3 | 1,228 | 32.4 | 1,218 | 31.4 | 1,144 | 30.1 | 1,223 | 31.5 |
| 1-2 conditions | 5,087 | 33.1 | 1,297 | 34.2 | 1,338 | 34.4 | 1,262 | 33.2 | 1,190 | 30.7 |
| 3+ conditions | 5,467 | 35.6 | 1,270 | 33.5 | 1,329 | 34.2 | 1,400 | 36.8 | 1,468 | 37.8 |
| **Frailty Index** | | | | | | | | | | |
| Not frail | 10,321 | 67.2 | 2,665 | 70.2 | 2,630 | 67.7 | 2,513 | 66.0 | 2,513 | 64.8 |
| Likely frail | 5,046 | 32.8 | 1,130 | 29.8 | 1,255 | 32.3 | 1,293 | 34.0 | 1,368 | 35.2 |

© 2024 Rahman SN et al. *JAMA Network Open*.

| | | | | | | | | | | |
|---|---|---|---|---|---|---|---|---|---|---|
| **Patient lives in metropolitan area** | | | | | | | | | | |
| No | 3,628 | 23.6 | 779 | 20.5 | 805 | 20.7 | 744 | 19.5 | 1,300 | 33.5 |
| Yes | 11,739 | 76.4 | 3,016 | 79.5 | 3,080 | 79.3 | 3,062 | 80.5 | 2,581 | 66.5 |
| **Dual Eligible** | | | | | | | | | | |
| No | 12,806 | 83.3 | 3,380 | 89.1 | 3,359 | 86.5 | 3,209 | 84.3 | 2,858 | 73.6 |
| Yes | 2,561 | 16.7 | 415 | 10.9 | 526 | 13.5 | 597 | 15.7 | 1,023 | 26.4 |
| **Limited Income Subsidy** | | | | | | | | | | |
| 100% Premium Subsidy, no copay | 701 | 4.5 | 136 | 4.5 | 153 | 4.5 | 167 | 4.5 | 245 | 4.5 |
| 100% Premium Subsidy, any Copay | 2,172 | 14.1 | 337 | 14.1 | 434 | 14.1 | 530 | 14.1 | 871 | 14.1 |
| 0-75% Premium Subsidy and copay | 12,494 | 81.3 | 3,322 | 81.3 | 3,298 | 81.3 | 3,109 | 81.3 | 2,765 | 81.3 |
| **Pre-Index Nephrectomy** | | | | | | | | | | |
| No | 13,142 | 85.5 | 3,193 | 84.1 | 3,340 | 86.0 | 3,258 | 85.6 | 3,351 | 86.3 |
| Yes | 2,225 | 14.5 | 602 | 15.9 | 545 | 14.0 | 548 | 14.4 | 530 | 13.7 |
| **Post-Index Nephrectomy** | | | | | | | | | | |
| No | 13,888 | 90.4 | 3,409 | 89.8 | 3,514 | 90.5 | 3,411 | 89.6 | 3,554 | 91.6 |
| Yes | 1,479 | 9.6 | 386 | 10.2 | 371 | 9.5 | 395 | 10.4 | 327 | 8.4 |
| **Survival After Index** | | | | | | | | | | |
| 31-60 days | 1,501 | 9.8 | 321 | 8.5 | 380 | 9.8 | 386 | 10.1 | 414 | 10.7 |
| 61-90 days | 1,097 | 7.1 | 261 | 6.9 | 286 | 7.4 | 254 | 6.7 | 296 | 7.6 |
| 91-180 days | 1,870 | 12.2 | 450 | 11.9 | 460 | 11.8 | 469 | 12.3 | 491 | 12.7 |
| 181-365 days | 2,129 | 13.9 | 533 | 14.0 | 487 | 12.5 | 549 | 14.4 | 560 | 14.4 |
| >365 days | 8,770 | 57.1 | 2,230 | 58.8 | 2,272 | 58.5 | 2,148 | 56.4 | 2,120 | 54.6 |

© 2024 Rahman SN et al. *JAMA Network Open*.

**eTable 3** – Oral Anticancer Agent (OAA) Treatment vs. None –stratified by Centers for Disease Control Social Vulnerability Index (CDC SVI) quartile

| Characteristic | Receipt of OAA treatment, Adjusted Relative Risk Ratio (95% Confidence Interval) | | | |
|---|---|---|---|---|
| | **Quartile 1 (Least Vulnerable)** | **Quartile 2** | **Quartile 3** | **Quartile 4 (Most Vulnerable)** |
| **Unadjusted percent who receive OAA treatment** | **24%** | **23%** | **22%** | **25%** |
| **Patient Race and Ethnicity (v. Non-Hispanic White)** | | | | |
| American Indian / Alaska Native, Other, Unknown | 0.88 (0.54-1.45) | 1.41 (0.84-2.35) | 0.84 (0.5-1.4) | 1.48 (0.91-2.4) |
| Asian/Pacific Islander | 1.8 (0.89-3.64) | 1.1 (0.64-1.89) | 1.39 (0.64-3.02) | 1.03 (0.56-1.91) |
| Hispanic | 0.76 (0.37-1.56) | 1.35 (0.82-2.22) | 1.45 (0.96-2.2) | 1.67 (1.28-2.18) |
| Non-Hispanic Black | 1.24 (0.79-1.96) | 0.58 (0.35-0.97) | 0.65 (0.47-0.89) | 0.82 (0.62-1.08) |
| **Dual Eligible** | 0.92 (0.46-1.84) | 0.91 (0.48-1.73) | 0.81 (0.48-1.39) | 0.93 (0.55-1.57) |
| **Limited Income Subsidy (vs. 0-75% Premium Subsidy)** | | | | |
| 100% Premium Subsidy, no copay | 0.94 (0.42-2.13) | 0.78 (0.36-1.69) | 0.88 (0.44-1.73) | 0.56 (0.29-1.06) |
| 100% Premium Subsidy, any copay | 1 (0.53-1.9) | 1.39 (0.76-2.54) | 1.2 (0.73-1.96) | 1.1 (0.66-1.82) |
| **Year of metastatic diagnosis (vs. 2015)** | | | | |
| 2016 | 0.84 (0.65-1.1) | 0.79 (0.6-1.03) | 0.83 (0.63-1.09) | 0.97 (0.75-1.26) |
| 2017 | 1.14 (0.87-1.49) | 1.1 (0.84-1.45) | 1.04 (0.78-1.37) | 1.19 (0.91-1.55) |
| 2018 | 0.94 (0.71-1.25) | 0.98 (0.74-1.29) | 0.87 (0.64-1.17) | 0.96 (0.72-1.27) |
| 2019 | 0.49 (0.35-0.67) | 0.45 (0.32-0.61) | 0.49 (0.35-0.68) | 0.52 (0.37-0.71) |
| **Age at diagnosis (vs. 66-70)** | | | | |
| 71-75 | 0.9 (0.72-1.13) | 0.91 (0.73-1.13) | 0.87 (0.69-1.08) | 0.88 (0.72-1.09) |
| 76-80 | 0.9 (0.71-1.14) | 0.82 (0.65-1.03) | 0.78 (0.62-0.98) | 0.74 (0.58-0.93) |
| 81+ | 0.49 (0.39-0.62) | 0.42 (0.33-0.53) | 0.33 (0.25-0.42) | 0.39 (0.31-0.5) |
| **Female (vs. Male)** | 0.69 (0.58-0.82) | 0.66 (0.56-0.79) | 0.8 (0.67-0.96) | 0.82 (0.7-0.97) |
| **Comorbidity score (vs. No conditions)** | | | | |
| 1-2 conditions | 0.87 (0.69-1.09) | 0.88 (0.7-1.1) | 1.05 (0.83-1.33) | 0.95 (0.75-1.19) |
| 3+ conditions | 0.91 (0.7-1.19) | 0.83 (0.64-1.09) | 0.89 (0.67-1.18) | 0.83 (0.64-1.08) |
| **Likely frail** | 0.84 (0.67-1.05) | 0.79 (0.63-0.99) | 0.83 (0.66-1.04) | 0.78 (0.63-0.97) |
| **Patient lives in metropolitan area** | 0.95 (0.78-1.17) | 0.99 (0.81-1.21) | 0.99 (0.8-1.22) | 0.94 (0.79-1.12) |

© 2024 Rahman SN et al. *JAMA Network Open*.

**eTable 4** – Immunotherapy (IO) Treatment vs. None –stratified by Centers for Disease Control Social Vulnerability Index (CDC SVI) quartile

| Characteristic | Receipt of IO treatment, Adjusted Relative Risk Ratio (95% Confidence Interval) | | | |
|---|---|---|---|---|
| | **Quartile 1 (Least Vulnerable)** | **Quartile 2** | **Quartile 3** | **Quartile 4 (Most Vulnerable)** |
| **Unadjusted percent who receive IO treatment** | **17%** | **17%** | **16%** | **16%** |
| **Patient Race and Ethnicity (v. Non-Hispanic White)** | | | | |
| American Indian / Alaska Native, Other, Unknown | 1.09 (0.63-1.89) | 0.87 (0.47-1.64) | 0.80 (0.44-1.48) | 1.17 (0.63-2.16) |
| Asian/Pacific Islander | 1.12 (0.44-2.84) | 1.14 (0.61-2.14) | 1.95 (0.82-4.64) | 0.81 (0.35-1.88) |
| Hispanic | 0.75 (0.29-1.91) | 0.89 (0.43-1.84) | 1.44 (0.86-2.42) | 1.00 (0.69-1.45) |
| Non-Hispanic Black | 0.96 (0.52-1.8) | 0.82 (0.47-1.41) | 0.61 (0.4-0.93) | 0.81 (0.57-1.15) |
| **Dual Eligible** | 1.6 (0.62-4.13) | 2.91 (0.81-10.38) | 0.47 (0.23-0.96) | 0.66 (0.33-1.29) |
| **Limited Income Subsidy (vs. 0-75% Premium Subsidy)** | | | | |
| 100% Premium Subsidy, no copay | 0.15 (0.04-0.55) | 0.04 (0.01-0.21) | 0.55 (0.20-1.50) | 0.39 (0.16-0.96) |
| 100% Premium Subsidy, any copay | 0.47 (0.02-1.13) | 0.24 (0.07-0.83) | 0.87 (0.46-1.64) | 0.88 (0.46-1.66) |
| **Year of metastatic diagnosis (vs. 2015)** | | | | |
| 2016 | 2.29 (1.41-3.72) | 2.42 (1.47-3.96) | 1.95 (1.20-3.19) | 1.93 (1.17-3.19) |
| 2017 | 2.82 (1.73-4.58) | 3.45 (2.11-5.63) | 3.36 (2.10-5.38) | 3.83 (2.39-6.15) |
| 2018 | 8.41 (5.33-13.26) | 10.29 (6.51-16.25) | 9.05 (5.79-14.16) | 8.07 (5.14-12.67) |
| 2019 | 15.38 (9.85-24.03) | 15.57 (9.94-24.39) | 12.93 (8.33-20.06) | 16.43 (10.55-25.57) |
| **Age at diagnosis (vs. 66-70)** | | | | |
| 71-75 | 0.86 (0.66-1.11) | 0.93 (0.72-1.2) | 0.88 (0.68-1.14) | 1 (0.77-1.3) |
| 76-80 | 0.79 (0.6-1.04) | 0.75 (0.57-0.98) | 0.76 (0.57-1.00) | 0.93 (0.7-1.23) |
| 81+ | 0.36 (0.27-0.48) | 0.43 (0.32-0.56) | 0.36 (0.27-0.49) | 0.56 (0.42-0.75) |
| **Female** | 0.65 (0.53-0.80) | 0.77 (0.63-0.94) | 0.78 (0.64-0.96) | 0.68 (0.55-0.84) |
| **Comorbidity score (vs. No conditions)** | | | | |
| 1-2 conditions | 1.13 (0.87-1.48) | 0.81 (0.63-1.04) | 0.85 (0.65-1.11) | 0.81 (0.61-1.07) |
| 3+ conditions | 0.83 (0.61-1.14) | 0.53 (0.39-0.72) | 0.79 (0.58-1.08) | 0.79 (0.58-1.08) |
| **Likely frail** | 0.75 (0.59-0.97) | 0.77 (0.60-0.98) | 0.73 (0.57-0.93) | 0.70 (0.55-0.90) |
| **Patient lives in metropolitan area** | 0.78 (0.62-1.00) | 1.13 (0.89-1.43) | 0.85 (0.66-1.08) | 0.86 (0.7-1.06) |

© 2024 Rahman SN et al. *JAMA Network Open*.

## eReferences


1. Eicheldinger C, Bonito A. More accurate racial and ethnic codes for Medicare administrative data. *Health Care Financ Rev*. Spring 2008;29(3):27-42.
2. Menendez ME, Neuhaus V, van Dijk CN, Ring D. The Elixhauser comorbidity method outperforms the Charlson index in predicting inpatient death after orthopaedic surgery. *Clinical orthopaedics and related research*. Sep 2014;472(9):2878-86. doi:10.1007/s11999-014-3686-7
3. Kim DH, Schneeweiss S, Glynn RJ, Lipsitz LA, Rockwood K, Avorn J. Measuring Frailty in Medicare Data: Development and Validation of a Claims-Based Frailty Index. *J Gerontol A Biol Sci Med Sci*. Jun 14 2018;73(7):980-987. doi:10.1093/gerona/glx229
4. Li P, Wong YN, Jahnke J, Pettit AR, Doshi JA. Association of high cost sharing and targeted therapy initiation among elderly Medicare patients with metastatic renal cell carcinoma. *Cancer Med*. Jan 2018;7(1):75-86. doi:10.1002/cam4.1262
5. Chow RD, Long JB, Hassan S, et al. Disparities in immune and targeted therapy utilization for older US patients with metastatic renal cell carcinoma. *JNCI Cancer Spectr*. May 2 2023;7(3)doi:10.1093/jncics/pkad036
6. Adepoju OE, Kiaghadi A. Measuring Historic and Longitudinal Social Vulnerability in Disaster-Prone Communities: A Modification to the Centers for Disease Control and Prevention Social Vulnerability Index (CDC-SVI). *Disaster Med Public Health Prep*. Feb 20 2023;17:e368. doi:10.1017/dmp.2023.29
7. Saelee R, Chandra Murthy N, Patel Murthy B, et al. Minority Health Social Vulnerability Index and COVID-19 vaccination coverage - The United States, December 14, 2020-January 31, 2022. *Vaccine*. Mar 17 2023;41(12):1943-1950. doi:10.1016/j.vaccine.2023.02.022
8. Social Deprivation Index (SDI). Robert Graham Center - Policy Studies in Family Medicine & Primary Care. Updated November 5, 2018. Accessed August 2, 2023,
9. Krieger N, Feldman JM, Waterman PD, Chen JT, Coull BA, Hemenway D. Local Residential Segregation Matters: Stronger Association of Census Tract Compared to Conventional City-Level Measures with Fatal and Non-Fatal Assaults (Total and Firearm Related), Using the Index of Concentration at the Extremes (ICE) for Racial, Economic, and Racialized Economic Segregation, Massachusetts (US), 1995-2010. *J Urban Health*. Apr 2017;94(2):244-258. doi:10.1007/s11524-016-0116-z


© 2024 Rahman SN et al. *JAMA Network Open*.
