# Supplementary material for: Area Vulnerability and Disparities in Therapy for Patients With Metastatic Renal Cell Carcinoma
Source: JAMA Netw Open. 2024 Apr 30;7(4):e248747. doi: 10.1001/jamanetworkopen.2024.8747 (PMC11061765; doi:10.1001/jamanetworkopen.2024.8747)
Supplement: Supplement 2. — Data Sharing Statement [file jamanetwopen-e248747-s002.pdf]

Data Sharing Statement

Rahman. Area Vulnerability and Disparities in Therapy for Patients With Metastatic Renal Cell Carcinoma. *JAMA Netw Open*. Published April 30, 2024.
doi:10.1001/jamanetworkopen.2024.8747

Data
Data available: No
